# SWATH-MS based quantitative proteomics analysis reveals novel proteins involved in PAMP triggered immunity against potato late blight pathogen *Phytophthora infestans*

**DOI:** 10.3389/fpls.2022.1036637

**Published:** 2022-11-18

**Authors:** Yang Mu, Xiao Guo, Jian Yu, Ruxun Wang, Zeng Liu, Kefan Hu, Jingyi Song, Lin Chen, Botao Song, Juan Du

**Affiliations:** ^1^ Key Laboratory of Horticultural Plant Biology, Ministry of Education, Huazhong Agricultural University, Wuhan, China; ^2^ Key Laboratory of Potato Biology and Biotechnology, Ministry of Agriculture and Rural Affairs, Huazhong Agricultural University, Wuhan, China; ^3^ College of Life Science and Technology, Huazhong Agricultural University, Wuhan, China

**Keywords:** *Nicotiana benthamiana*, *Phytophthora infestans*, pathogen-associated molecular pattern, immunity, SWATH-MS

## Abstract

Potato is the most important non-grain food in the world, while late blight caused by *Phytophthora infestans* seriously threatens the production of potato. Since pathogen-associated molecular patterns (PAMPs) are relatively conserved, PAMP-triggered immunity (PTI) can provide durable resistance to late blight for potato. However, knowledge of the regulatory mechanisms of PTI against oomycete pathogens at protein levels remains limited due to the small number of identified proteins. In the present work, changes in the proteome profile of *Nicotiana benthamiana* leaves upon *P. infestans* PAMP induction were examined using the SWATH-MS (sequential windowed acquisition of all theoretical mass spectra) approach, which provides quantification of protein abundances and large-scale identification of PTI-related proteins. A total of 4401 proteins have been identified, of which 1429 proteins were differentially expressed at least at one time point of 8, 12, 24 and 48 h after PAMP induction, compared with the expression at 0 h when immediately after PAMP induction. They were further analyzed by expression clustering and gene ontology (GO) enrichment analysis. Through functional verification, six novel DEPs of 19 candidates were proved to be involved in PTI responses, including mitochondrial phosphate carrier protein (MPT) 3, vesicle-associated membrane protein (VAMP) 714, lysophospholipase (LysoPL) 2, ascorbate peroxidase (APX) 1, heat shock 70 kDa protein (HSP) 2 and peptidyl-prolyl cis-trans isomerase FKBP (FKBP) 15-1. Taken together, the time course approach and the resulting large-scale proteomic analyses have enlarged our understanding of PTI mechanisms and provided a valuable resource for the discovery of complex protein networks involved in the resistance response of potato to late blight.

## Introduction

Potato is the most important non-grain food in the world. However, it severely suffers from late blight, which is caused by the devastating oomycete pathogen *Phytophthora infestans*. Enhancing the resistance of potato to *P. infestans* is the most economical and environmentally friendly approach to control late blight. Plant immune systems rely on membrane-resident pattern recognition receptors (PRRs) and intracellular nucleotide-binding oligomerization domain-like receptors (NLRs) to recognize and prevent pathogen invasion (Couto and Zipfel, 2016; Jones et al., 2016). PRR-mediated resistance is considered to be relatively more durable.

Plant PRRs can recognize conserved pathogen-associated molecular patterns (PAMPs), such as bacterial flagellin or its derived peptide, flg22, to provide broad-spectrum pathogen resistance (Zipfel, 2008; Couto and Zipfel, 2016). The experiment system of flg22-triggered reactive oxygen species (ROS) bursts has been well established and widely used for the study of PAMP-triggered immunity (PTI) (Sang and Macho, 2017). Elicitins are the most well-characterized oomycete PAMPs specifically secreted by *Phytophthora* and *Pythium* species, which share a highly conserved 98-amino-acid domain (Pfam PF00964) (Ponchet et al., 1999; Jiang et al., 2006; Lévesque et al., 2010; Derevnina et al., 2016). The receptor and co-receptors of elicitins have previously been identified, namely elicitin response (ELR), somatic embryogenesis receptor kinase (SERK) 3/brassinosteroid-associated kinase (BAK) 1 and suppressor of bir1-1 (SOBIR1) (Du et al., 2015; Domazakis et al., 2018). Besides, some other genes such as *SGT1* (Shibata et al., 2011), *SIPK/NTF6* (Asai et al., 2008), *WRKY7/8/9/11* (Adachi et al., 2015), *NbMKK1* (Takahashi et al., 2007), *Nbrboh* (Yoshioka et al., 2003) and *HSP90* and *HSP70* (Kanzaki et al., 2003) have been proved important for elicitin-triggered immunity. Moreover, we have identified 32 differentially expressed proteins (DEPs) in *Nicotiana benthamiana* induced by elicitin through iTRAQ proteomics approach and proved two DEPs namely, ATP dependent transporter and 60S ribosomal protein L15, are essential for PAMP-triggered immunity (Du et al., 2017). However, the mechanism of PTI against *P. infestans* still needs further exploration.

Compared with iTRAQ, another recently developed label-free quantitative proteomics approach named SWATH-MS (sequential windowed acquisition of all theoretical fragment ions - mass spectra) is based on a data-independent acquisition (DIA) strategy for consistent and reproducible quantification of peptides with high complexity, and thus has high coverage, unlimited sample size and can quantify more low abundance proteins (Huang et al., 2015). In this study, SWATH-MS approach was used to screen proteins involved in PTI against *P. infestans*. The culture filtrate (CF) of *P. infestans* contains a variety of PAMPs including elicitins and so on (McLellan et al., 2013; Saubeau et al., 2014). We did a time course analysis of protein abundance in *N. benthamiana* leaves at 0, 8, 12, 24 and 48 h after CF infiltration. A total of 4401 proteins were quantitatively identified by SWATH-MS in *N. benthamiana* leaves, 1429 of which were found to be differentially expressed by comparing the amount of identified proteins at the later four time points with that of 0 h post infiltration (hpi) at a fold change of 1.5 with a *p*-value <0.05. According to bioinformatic analysis results, 19 DEPs belonging to the gene ontology (GO) term of “response to stress” were selected for further functional verification by virus-induced gene silencing (VIGS) and PAMP-triggered reactive oxygen species (ROS) burst analysis. As a result, six novel genes were found involved in PTI responses.

## Methods

### *Phytophthora infestans* culture filtrate preparation

For preparing culture filtrate (CF), the mycelia and sporangia of a *P. infestans* isolate 88069 were cultured in the Plich liquid medium (0.5 g KH_2_PO_4_, 0.25 g MgSO_4_·7H_2_O, 1 g Asparagine, 1 mg Thiamine, 0.5 g Yeast extract, 10 mg β-sitosterol and 25 g Glucose in 1l) for four weeks at 16°C in dark. Then the liquid was used for plant treatment.

### Plant materials and treatments

*N. benthamiana* plants were grown from seeds and maintained in the controlled climate chamber under a 16h/8 h light and dark cycle at 24°C. The fully expanded leaves of 4-5-week-old plants were used for CF infiltration with a needleless syringe. And samples were taken at 0 h (immediately after CF infiltration), 8 h, 12 h, 24 h, and 48 h, respectively. Three infiltrated leaves were taken at each time point as three biological replicates.

### Protein and peptide preparation

Approximately 1.5 g of *N. benthamiana* leaves for each sample was ground in a mortar with liquid nitrogen. The tissues were homogenized by 10 ml of SDS Lysis Buffer (60 g sucrose, 4 g SDS and 3.084 g DTT in 200 ml) containing protease inhibitor and centrifuged at 20 000 rpm at 4°C for 10 min. The supernatant was taken to a freshly pre-cooled 50 ml centrifuge tube and mixed with an equal volume of Tris saturated phenol (pH 8.0). Afterward, the mixture was incubated at 37°C on a shaker for 5 min. After centrifugation at 20 000 rpm at 4°C for 10 min, the mixture was divided into three layers, with protein in the upper layer. It was then carefully taken out and mixed with 5× volumes of pre-cooled 0.1 M ammonium acetate in methanol for overnight protein precipitation at -20°C. After centrifuged at 12 000 rpm for 10 min at 4°C, the supernatants were removed, and the pellets were washed with 10 ml of pre-cooled methanol, and then further washed with 10 ml of pre-cooled acetone. Finally, the protein pellets were dissolved in the 4 ml urea buffer (8 M urea in 0.05 M ammonium bicarbonate) with ultrasonic for 6 min (ultrasonic for 2 s, stop for 3 s). After centrifugation at 20 000 rpm for 10 min at 4°C, the supernatant was carefully aspirated into a freshly pre-cooled 15 ml centrifuge tube for subsequent protein quantification. Protein concentration was determined using the Pierce™ bicinchoninic acid (BCA) Protein Assay Kit (Thermo Scientific, USA) with bovine serum albumin (BSA; 2mg/ml) as a standard for quantification.

Then each protein sample (5 mg) was reduced by 20 μl of 1 M dithiothreitol (DTT) at 56°C for 30 min and alkylated by 120 μl of 0.5 M iodoacetamide at room temperature in dark for 40 min. Then 40 μl of 1 M DTT was added into the protein materials in dark for 20 min to quench the extra iodoacetamide. Afterward, each sample was diluted into 8× volumes of 50 mM ammonium bicarbonate solution including 1 M urea and 400 μl of trypsin (enzyme/protein, 1:25 w/w) for protein digestion overnight at 37°C. Peptides were generated by specific trypsin cleavage at the C-terminal at the arginine and lysine sites of proteins. The acquired peptides were acidified by 10% trifluoroacetic acid (TCA) and desalted using oasis^®^ HLB solid-phase extraction (SPE; Agilent, USA). Finally, the desalted peptide samples were dried in a speed-vacuum concentrator and stored at -80°C or directly dissolved in 0.1% formic acid for LC-MS/MS analysis.

### SWATH-MS measurement and analysis

SCX peptide fractionation was performed according to previously reported (Lu et al., 2019). Analysis of SCX peptide fractions was performed on a Nanospray III source and a TripleTOF 5600+ (AB SCIEX) mass spectrometer (Andrews et al., 2011). The peptide fractions (2μg each) were resuspended in 0.1% formic acid (FA) and trapped separately into an Ekspert TM nanoLC 415 (AB SCIEX) with a nanoLC trap (ChromXP C18-CL 3 μm 120 Å, 350μm × 0.5 mm) at 2 μL/min for 7 min. Then, the pump flow was split to obtain a flow rate of 300 nL/min on the nanoLC trap and the nanoLC column (75 μm × 15 cm, 3C18-CL-120, 3 μm, 120 Å) was used for separation. The mobile phases consisted of solvent A containing water: acetonitrile: formic acid at a ratio of 98:2:0.1 (V/V/V), and solvent B containing water: acetonitrile: formic acid at a ratio of 2:98:0.1 (V/V/V). A nonlinear gradient of 5% to 12% B for 0.5 min, 12% to 24% B for 59.5 min, 24% to 35% B for 35 min, 35% to 50% B for10 min, 50% to 80% B for 1 min, 80% B for 5min, 80% to 5% B in 1 min, and 5% B for 8 min was employed. The mass tolerance was set to 50mDa, The spray voltage was set to 2.5 kV, and the temperature of the heated capillary was set to 275°C. The mass spectrometer was operated in positive ion mode with a nanoion spray voltage of 2.5 kV.

Data-dependent acquisition (DDA) was first performed to obtain the SWATH-MS spectral ion library. A survey scan of 0.248 s in the range 350−1250 m/z was performed to collect the MS1 spectra. The top 50 precursor ions with charge state from +2 to +5 were selected for fragmentation with an accumulation time of 50 ms per MS/MS experiment for a total cycle time of 2.3 s, and MS/MS spectra were collected in the range 100−1500 m/z. Selected ions and their isotopes were dynamically excluded from further MS/MS fragmentation for 18 s.

In the SWATH analysis, the same peptide samples were analyzed by the cyclic data-independent acquisition (DIA) mode using similar methods as described above. For DIA analysis, MS/MS proteome profiling was analyzed by the same LC-MS/MS system. A survey scan of 50 ms was performed and all precursors were subjected to fragmentation. MS/MS experiments were conducted with an accumulation time of 100 ms per 25 Da swath (total swath 36) for a total cycle time of 3.6 s.

All DDA mass spectrometry data were used to generate a reference spectral library by searching against the *Nicotiana benthamiana* protein database v04.4 (36151 proteins, 2010 release, Sol Genomics Network) using the ProteinPilot 4.5 software (Sciex). The parameters included digestion, alkylation, and biological modification. A false discovery rate of < 1% was accepted as the criteria for the peptide assignments and protein identification. Subsequently, the DIA data and spectral library were loaded into PeakView v.1.2 software (Sciex) under restricted criteria and settings: six peptides, six transitions, 99% peptide confidence and ion library mass tolerance (75 ppm). The output of quantified proteins and corresponding peptides by the MarkerView (Sciex) generated three quantitative files including the extracted peak area for individual fragment ions, the sum of the fragment ion areas for each peptide and the sum of peptide areas for each protein. Differentially expressed proteins (DEPs) were analyzed by ANOVA analysis and Tukey’s HSD multiple comparison (*P* ≤ 0.05) and then were updated from reference genome Niben v0.44 to Niben v1.01. Information of the identified DEPs were shown in **Table S1**.

### Gene ontology enrichment analysis

TBtools (Chen et al., 2020) was used to perform gene ontology (GO) enrichment for DEPs of cluster 7. GO background data was downloaded from the AgriGO website. E-value cut-off: 1.0E-3, and GO terms of Biological Process with a false discovery rate (FDR) 0.05 were obtained and included in **Table S2**. They were ranked according to their *P*-values (ascending), and the top 15 terms for Biological Process belonging to level 3 were then selected to draw a bubble diagram by R package ggplot2 (https://github.com/tidyverse/ggplot2).

### Tobacco rattle virus-induced gene silencing

Virus-induced gene silencing (VIGS) constructs were made by cloning ∼300 bp PCR fragments from candidate genes into the TRV2 vector, respectively (Ratcliff et al., 2001). The inserted fragments were selected with the help of a VIGS tool in the genome website of Solanaceae (https://vigs.solgenomics.net/). Primers for VIGS of candidate genes shown in **Table S3**. A TRV2 construct expressing GFP was used as a control (Du et al., 2017). *Agrobacterium tumefaciens* strains GV3101 containing TRV1 and each candidate gene VIGS construct at an OD600 of 0.3 were mixed at a 1:1 ratio and then infiltrated into the leaves of the four-leaf-stage *N. benthamiana* plant. Systemic leaves were detached and analyzed by qRT-PCR.

### Quantitative RT-PCR analysis

Total RNA was extracted from the leaves of *N. benthamiana* plants after infiltration of CF and VIGS using a Total RNApure Kit (Zoman Biotechnology Co. Ltd.) according to the manufacturer’s instructions. First-strand cDNA was synthesized using the 5X All-in-One RT MasterMix Reverse Transcription Kit (ABM). qRT-PCR was carried out using SYBR green as described previously (Du et al., 2017). Primers for qRT-PCR are shown in **Table S3** and *Actin* was used as an internal control. For VIGS experiment, at least one primer was designed outside the region of cDNA targeted for silencing.

### ROS analysis

Luminol-based assays were used to evaluate ROS bursts. After three weeks of VIGS, at least nine leaves of *N. benthamiana* plants for each candidate gene were excised into leaf discs of 0.09 cm^2^. To eliminate the wounding effect, leaf discs were incubated in the 96-well plate with 200 µl of ddH_2_O overnight, which was replaced by 100 µl of reaction solution as previously described with a difference in the concentration of flg22 (Sang and Macho, 2017). Instead of 5 μl flg22 stock solution (100 μM) added to 10 ml of ultra-pure distilled H_2_O, 10 μl flg22 stock solution (100 μM) was added in this study. Measurements were taken immediately after adding the solution with a 1 min interval reading time for a period of 60 min with a luminometer. The measurement values for ROS production from 24 leaf discs per treatment were indicated as means of RLU (Relative Light Units).

## Results

### *Phytophthora infestans* culture filtrate induces cell death in *Nicotiana benthamiana* within 48 h

**Figure 1:**
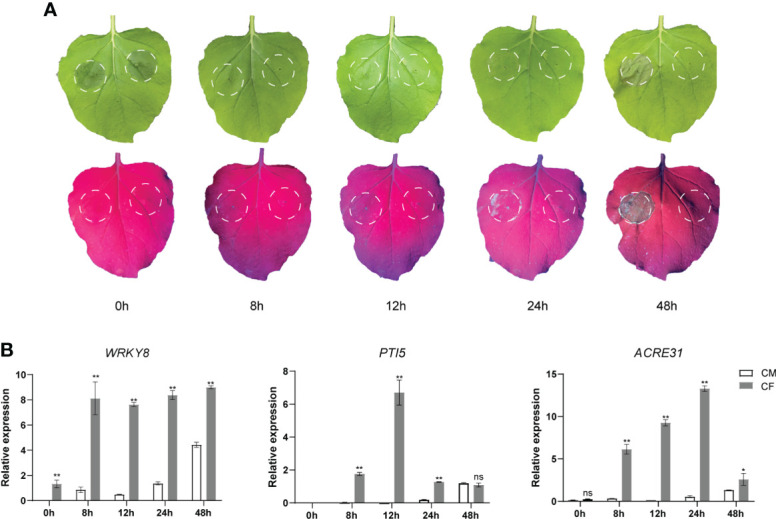
The culture filtrate (CF) of *Phytophthora infestans* induced PAMP triggered immunity responses in *Nicotiana benthamiana.* **(A)** CF was infiltrated within a white line circle on the left panel of the mid vein while culture medium (CM) was on the right panel. **(B)** CF-induced upregulation of PTI marker genes *WRKY8*, *PTI5* and *ACRE31.* Independent experiments have been conducted three times with similar results. Student’s *t*-test was used for the statistical analysis. Error bars stand for SEM, ns, not significant, *P < 0.05 and **P < 0.01.

To test the PAMP-triggered immune responses by *P. infestans* culture filtrate (CF), it as well as a negative control culture medium (CM) were infiltrated into the same *N. benthamiana* leaves, respectively. Then the infiltrated leaves were observed and sampled at 0 (immediately after CF infiltration), 8, 12, 24 and 48 hpi. Under natural light, no obvious phenotypes were observed at 0, 8, 12 and 24 hpi, while at 48 hpi, a cell death was visible at the CF-infiltrated site but not at the CM-infiltrated site (**Figure 1A**). Interestingly, under UV light, little cell death responses could be observed as early as at 24 hpi at the CF-infiltrated site but not at the CM-infiltrated site (**Figure 1A**). Then samples taken at 0, 8, 12, 24 and 48 hpi were analyzed for the expression of three PTI marker genes *WRKY8, PTI5* and *ACRE31* (Nguyen et al., 2010; Wang et al., 2018) by qPCR. The expression of all three genes was significantly upregulated at 8 h after CF infiltration, however, their expression changes were diverse during 48 hpi (**Figure 1B**). After 8 hpi, the expression of *WRKY8* kept steady until 48 hpi, while the expression of *PTI5* and *ACRE31* peaked at 12 and 24 hpi, respectively, and then fell down (**Figure 1B**).

### CF induced a total of 4401 proteins at five time points

**Figure 2:**
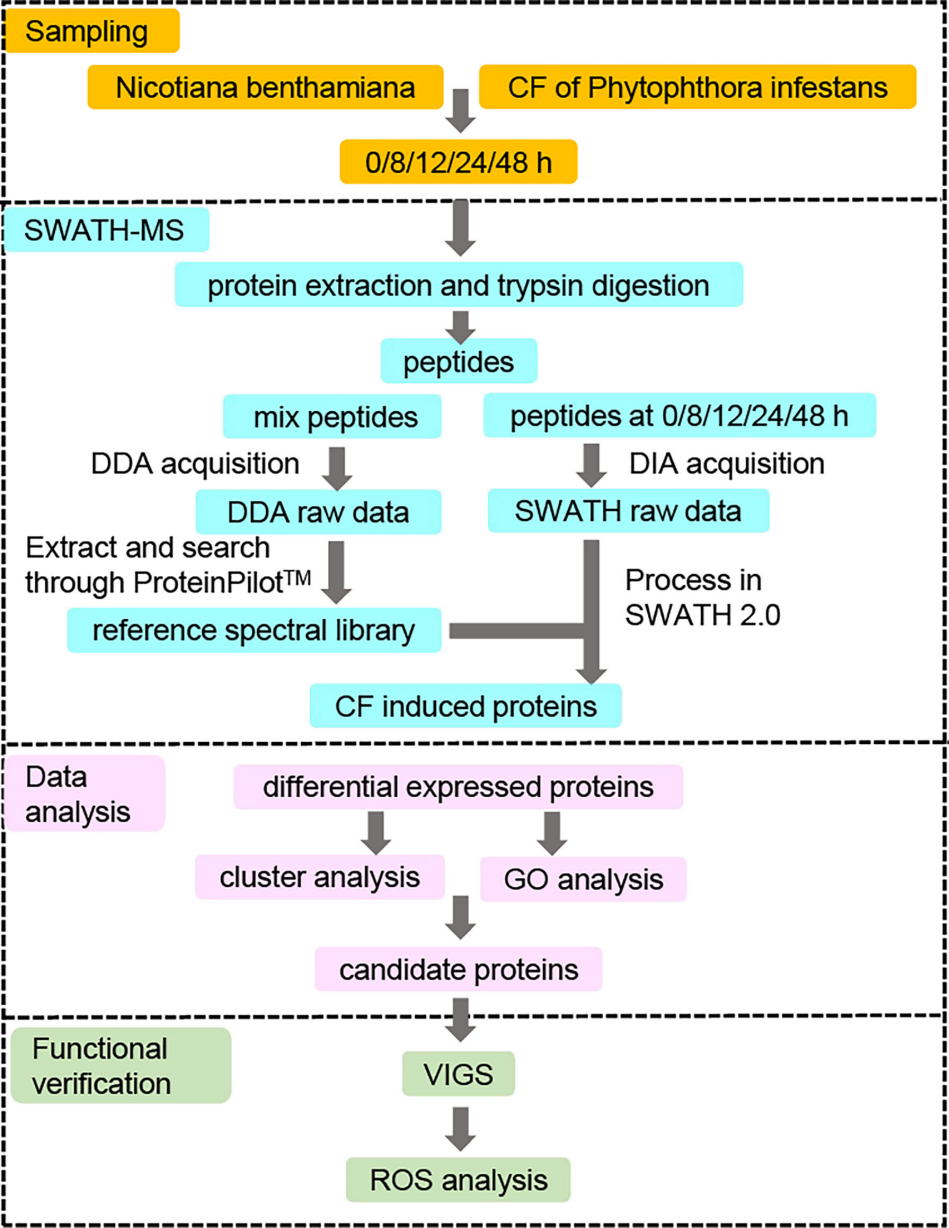
Strategy for identifying novel differentially expressed proteins (DEPs) involved in PAMP triggered immunity against *Phytophthora infestans*. *N. benthamiana* leaves were infiltrated with culture filtrate (CF) of *P. infestans*, and samples were collected at 0 (immediately after infiltration), 8, 12, 24 and 48 hpi. Proteins were extracted from the samples at five time points and digested by trypsin for the SWATH-MS analysis. The resulting peptides were first mixed and measured by a data-dependent acquisition (DDA) model to generate a reference spectral library. At the same time, the resulting peptides at each of the five time points were also measured by a data-independent acquisition (DIA) model to obtain SWATH raw data, which were compared to the reference spectral library to get the CF-induced proteins. Differentially expressed proteins (DEPs) were identified by comparing the expression of CF-induced proteins at 8, 12, 24 and 48 hpi to that at 0 hpi at a fold change of 1.5 with a *p*-value <0.05. Then protein expression clustering and GO enrichment analysis were performed to get the candidate proteins for the functional verification by virus-induced gene silencing (VIGS) and reactive oxygen species (ROS) assays.

In order to identify novel differentially expressed proteins (DEPs) induced by CF in *N. benthamiana*, only CF-infiltrated leaves taken at 0, 8, 12, 24 and 48 h were sent for the subsequent SWATH-MS analysis (**Figure 2**). For each time point, three infiltrated leaves were taken for independent tests and each sample was also tested three times for SWATH-MS as technical repeats. As a result, nine data sets were obtained for each time point. With data from all the five time points, a reference database containing a total of 38,683 unique peptides corresponding to 5714 proteins was generated using the data-dependent acquisition (DDA) model, with a false discovery rate (FDR) less than 1%. Compared with our reference database, a total of 4401 proteins were identified at all five time points with the Process in SWATH 2.0 software analysis based on important information including retention time, precursor ion mass, and fragment ion mass-to-charge ratio.

### A total of 1429 DEPs were identified

**Figure 3:**
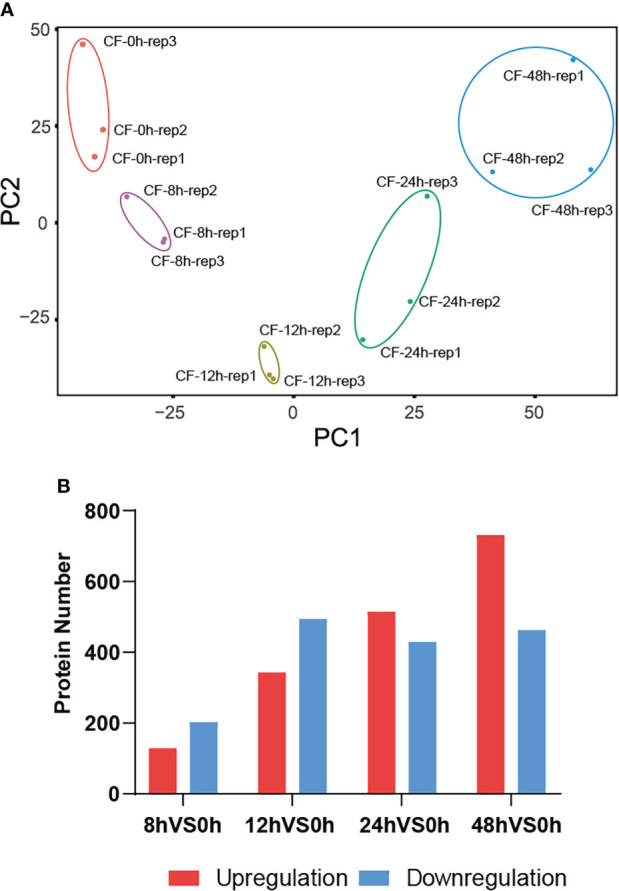
A total of 1429 DEPs were identified. The principal component analysis (PCA) biplot shows that CF-induced proteins at five time points 0, 8, 12, 24, 48 hpi were distinctly separate in two principal components **(A)**, and they were differentially expressed at 8, 12, 24, 48 hpi compared to their expression at 0 hpi **(B)**.

Then principal component analysis (PCA) was performed to evaluate the data by R package factominer. The idensity of all 4401 proteins identified at five time points were taken as observation values, and samples at different time points and their biological replicates were taken as variables. The PCA biplot shows that they were distinctly separate in two principal components (**Figure 3A**), which means that proteins has experienced a significant change in *N. benthamiana* after CF induction. Among the above identified 4401 proteins, we compared the amount of identified proteins at the later four time points with that of 0 hpi. A total of 1429 DEPs were identified at a fold change of 1.5 with a *p*-value <0.05 (**Table S1**). Specifically, 331 DEPs were identified with 129 up-regulated and 202 down-regulated at 8 hpi, 837 DEPs were identified with 343 up-regulated and 494 down-regulated at 12 hpi, 944 DEPs were identified with 515 up-regulated and 429 down-regulated at 24 hpi, 1193 DEPs were identified with 731 up-regulated and 462 down-regulated at 48 hpi (**Figure 3B**).

### Defense-related terms were found in Cluster 7

**Figure 4:**
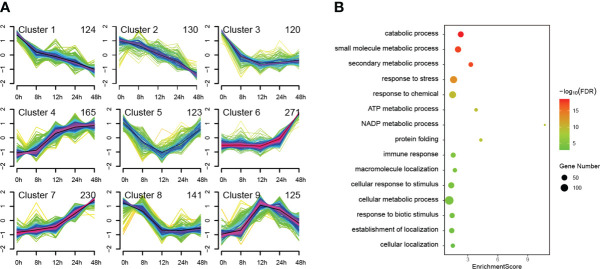
Cluster and GO enrichment analysis of 1429 differentially expressed proteins (DEPs). The identified 1429 DEPs were clustered in mainly nine types of temporal patterns according to their protein expression profiles **(A)** and GO enrichment analysis was performed on the DEPs of cluster 7, of which the top 15 GO terms for Biological Process belonging to level 3 are presented **(B)**.

We used Mfuzz to cluster all 1429 DEPs according to their protein expression profiles and obtained mainly nine types of temporal patterns, which generally show four expression trends (**Figure 4A**). The first trend is overall down-regulated shown in clusters 1, 2, 3 and 8. The second trend is overall upregulated shown in clusters 4, 6 and 7. The third trend is first down-regulated then up-regulated shown only in cluster 5. The fourth trend is first up-regulated then down-regulated shown only in cluster 9. Among the upregulated clusters, the trend of cluster 7 maintains the best continuity, which may be more correlated to PTI defenses. To further analyze the function of DEPs in cluster 7, we performed GO enrichment analysis of them (**Figure 4B**). Among the top 15 GO terms for Biological Process belonging to level 3, defense-related terms were found such as response to stress, immune response, response to biotic stimulus, etc. Interestingly, some well-known PTI-related proteins such as SGT1 and BAK1 were also found in cluster 7. This indicates that more PTI-related proteins may be involved in cluster 7 since they have similar expression patterns.

### Six novel candidate DEPs were identified to be involved in PTI responses

To test the above hypothesis, we selected 19 DEPs belonging to “response to stress” for functional verifications by VIGS and ROS assays, including mitochondrial phosphate carrier protein (MPT) 3, protein disulfide-isomerase like (PDIL) 2, peroxisomal (S)-2-hydroxy-acid oxidase (GOX) 3, cysteine-rich receptor-like protein kinase (CRK) 2, thioredoxin reductase (NTRA) 2, heat shock 70 kDa protein BIP2 (HSP70-12), chaperone protein dnaJ (DJA) 2, calnexin homolog (CNX) 1, lysophospholipase (LysoPL) 2, PLAT domain-containing protein (PLAT) 1, ascorbate peroxidase (APX) 1, 2-hydroxyacyl-CoA lyase (HPCL), ABC transporter G family member (ABCG) 40, acidic endochitinase (CHIA), early responsive to dehydration (ERD) 9, peptidyl-prolyl cis-trans isomerase FKBP (FKBP) 15-1, vesicle-associated membrane protein (VAMP) 714, heat shock 70 kDa protein (HSP) 2, nucleosome assembly protein (NAP) 1;3 (**Table 1**).

**Table 1:** Information of 19 candidate proteins in cluster 7.

Accession no.	Name	Length (aa)	Annotation
Niben101Scf00109g00017.1	MPT3	366	Mitochondrial phosphate carrier protein 3
Niben101Scf00332g04004.1	PDIL2	359	Protein disulfide-isomerase like 2-1
Niben101Scf00622g00006.1	GOX3	368	Peroxisomal (S)-2-hydroxy-acid oxidase
Niben101Scf01230g01011.1	CRK2	604	Cysteine-rich receptor-like protein kinase 2
Niben101Scf01677g04003.1	NTRA	330	Thioredoxin reductase 2
Niben101Scf02030g04003.1	HSP70-12	583	Heat shock 70 kDa protein BIP2
Niben101Scf03138g01010.1	DJA2	420	Chaperone protein dnaJ 2
Niben101Scf03777g00002.1	CNX1	539	Calnexin homolog 1
Niben101Scf04820g01003.1	LysoPL2	327	Lysophospholipase 2
Niben101Scf05326g00012.1	PLAT1	183	PLAT domain-containing protein 1
Niben101Scf06195g00002.1	APX1	250	Ascorbate peroxidase 1
Niben101Scf06394g00009.1	HPCL	513	2-hydroxyacyl-CoA lyase
Niben101Scf06583g03008.1	ABCG40	1422	ABC transporter G family member 40
Niben101Scf06684g03003.1	CHIA	295	Acidic endochitinase
Niben101Scf06958g02005.1	ERD9	216	Protein early responsive to dehydration 9
Niben101Scf08447g01002.1	FKBP15-1	117	Peptidyl-prolyl cis-trans isomerase FKBP15-1
Niben101Scf08515g00019.1	VAMP714	217	Vesicle-associated membrane protein 714
Niben101Scf08590g00005.1	HSP70-2	706	Heat shock 70 kDa protein 2
Niben101Scf11512g01019.1	NAP1;3	384	Nucleosome assembly protein 1;3

**Figure 5:**
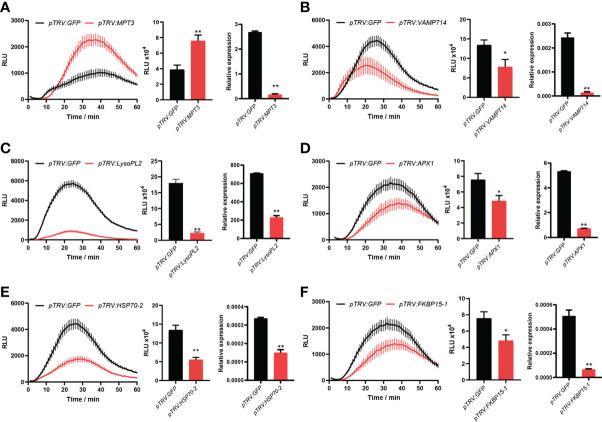
Six novel candidate DEPs were found involved in PTI responses. After virus-induced gene silencing (VIGS) of *MPT3* **(A)**, the ROS production was significantly up-regulated, while after VIGS of *VAMP714* **(B)**, *LysoPL2* **(C)**, *APX1* **(D)**, *HSP70-2* **(E)** and *FKBP15-1* **(F)**, the ROS production was significantly down-regulated. The bar charts show the total count of ROS accumulation and silencing efficiency of VIGS. Independent experiments have been conducted three times with similar results. Student’s *t*-test was used for the statistical analysis. Error bars stand for SEM, *P < 0.05 and **P < 0.01.

For VIGS, ∼300 bp PCR fragments from 19 candidate genes were amplified using the cDNA of *N. benthamiana* as a template. Leaves of four-leaf-stage plants were treated with TRV-candidate genes and the control TRV-*GFP*, respectively. After three weeks, three leaves from different plants of *N. benthamiana* for each candidate gene were excised, and flg22 was used to determine the ROS intensity. Compared to the control TRV-GFP, we found that the ROS production was significantly changed after plants were treated with six TRV-candidate genes. After VIGS of a candidate gene *MPT3*, the ROS production was significantly up-regulated, while after VIGS of five other candidate genes including *VAMP714*, *LysoPL2*, *APX1*, *HSP70-2* and *FKBP15-1*, the ROS production was significantly down-regulated (**Figure 5**). Their silencing efficiency was tested by qRT-PCR with the transcript level of candidate genes reduced below 50% (**Figure 5**).

## Discussion

The mechanism of PAMP triggered immunity (PTI) against oomycete pathogens is largely unknown. In this study, we have performed a label-free quantitative proteomics approach named SWATH-MS to screen proteins involved in PTI against *P. infestans* (**Figure 2**). The culture filtrate (CF) of *P. infestans* was used to provide PAMPs (McLellan et al., 2013; Saubeau et al., 2014). A total of 4401 proteins were induced in *Nicotiana benthamiana* at 0, 8, 12, 24 and 48 hpi. And 1429 proteins were differentially expressed at least at one time point of 8, 12, 24 and 48 hpi (**Figure 3**; **Table S1**). With the time course expression profiles, they could be divided into nine clusters (**Figure 4A**). Cluster 7 contains some defense-related GO terms including “response to stress” (**Figure 4B**). Then 19 DEPs in this term were selected as candidates for functional verification by VIGS and ROS assay. As a result, six DEPs were proved to be involved in PTI responses (**Figure 5**).

Previously, we used another proteomics approach iTRAQ to identify proteins involved in PTI against *P. infestans* in *N. benthamiana*, which resulted in only 32 DEPs induced by INF1 elicitin (Du et al., 2017 ). Compared with iTRAQ, SWATH-MS has higher coverage and better repeatability, no limit on sample size, and can quantify more low-abundance proteins (Huang et al., 2015). Thus, here we could detect samples at five time points, while only one time point at 8 hpi was available by iTRAQ. Besides, the inducers used in the two proteomics approaches are different. The INF1 elicitin was used for iTRAQ, while CF was used for SWATH-MS, which contains a variety of PAMPs including at least three elicitins e.g. INF1, INF4, INF5, and a galactan-based complex polysaccharide (Saubeau et al., 2014). Thus, CF could induce much more DEPs and complex PTI responses.

We validated the involvement of candidate DEPs with bacterial flg22-triggered ROS burst, which is not from *P. infestans* CF. However, they may trigger similar PTI signaling. Actually, CF infiltration and agroinfiltration of INF1 have also been performed on the VIGS plants of candidate DEPs. However, there was no obvious attenuation of cell death mediated by CF or INF1 (data not shown). We guess that they may play a negative role or have redundancy in the PTI response. The well-established flg22-triggered ROS bursts system on the VIGS plants could better quantify their influences on the PTI responses. Indeed, among the six identified DEPs, one plays a negative role and the other five partially affect the ROS bursts after treated with their corresponding TRV vectors compared with the controls.

The six identified DEPs include mitochondrial phosphate carrier protein (MPT) 3, vesicle-associated membrane protein (VAMP) 714, lysophospholipase (LysoPL) 2, ascorbate peroxidase (APX) 1, heat shock 70 kDa protein (HSP) 2 and peptidyl-prolyl cis-trans isomerase FKBP15-1. MPT3 plays a crucial role in ATP production in plant cells. In eukaryotes, N-ethylmaleimide-sensitive factor adaptor protein (SNAP) receptors, also known as SNAREs, have evolved as mediators of fusion of membranes between vesicles and targets. Arabidopsis AtVAMP714 is essential for the polarisation of PIN proteins and auxin responses (Gu et al., 2021), while rice OsVAMP714-mediated trafficking pathway was indicated to play an important role in rice blast resistance (Sugano et al., 2016). LysoPL2 is involved in tolerance to cadmium-induced oxidative stress and also reported to function as Caffeoyl shikimate esterase (CSE) that is an enzyme in the lignin biosynthetic pathway in Arabidopsis (Vanholme et al., 2013). CSE converts caffeoyl shikimate to caffiate and its mutants have reduced lignin content and collapsed vessel elements. APX1 plays an important role in the regulation of the steady-state level of ROS in plant cells (Mittler et al., 2004). HSP70 is an essential protein regulator involved in maintaining internal stability such as proper folding of proteins and breakdown of unfolded proteins. NbHSP70c-1 (GenBank no. AB105430) was previously reported to be essential for INF1-triggered cell death response (Kanzaki et al., 2003). HSP70-2 identified in this study shares ∼50% similarities of amino acids with NbHSP70c-1, which could play similar roles as NbHSP70c-1. FKBP15-2 was shown to be required for ER stress-mediated plant immunity (Fan et al., 2018). However, the mechanisms of them involved in PTI responses still needs further exploration.

Overall, our study sets an example to use SWATH-MS approach to study plant immunity. The six identified DEPs provide new clues to make a further deep study of the molecular mechanism of PTI. Moreover, the identified 1429 DEPs also provide a valuable resource for the discovery of complex protein networks involved in the resistance response of potato to late blight.

## Data availability statement

All data supporting the findings of this research are available within the paper and within its supplementary data published online.

## Author contributions

YM, XG, JY, RW, ZL, KH, JS, LC and JD performed experiments and analyzed data. YM, XG, JY, RW, ZL, KH, JS, BS and JD designed experiments. BS and JD supervised. YM, XG, LC and JD wrote the manuscript. All authors contributed to the article and approved the submitted version.

## Funding

The work was partially supported by the National Science Foundation of China (31401436), the Fundamental Research Funds for the Central Universities (2662016QD042) and the Key-Area Research and Development Program of Guangdong Province (2020B020219002).

## Acknowledgments

We thank Qiansi Chen for providing the SWATH-MS proteomics platform and guidance.

## Conflict of interest

The authors declare that the research was conducted in the absence of any commercial or financial relationships that could be construed as a potential conflict of interest.

## Publisher’s note

All claims expressed in this article are solely those of the authors and do not necessarily represent those of their affiliated organizations, or those of the publisher, the editors and the reviewers. Any product that may be evaluated in this article, or claim that may be made by its manufacturer, is not guaranteed or endorsed by the publisher.
